# TiO_2_ as a Photocatalyst for Water Splitting—An Experimental and Theoretical Review

**DOI:** 10.3390/molecules26061687

**Published:** 2021-03-17

**Authors:** Håkon Eidsvåg, Said Bentouba, Ponniah Vajeeston, Shivatharsiny Yohi, Dhayalan Velauthapillai

**Affiliations:** 1 Department of Computing, Mathematics and Physics, Western Norway University of Applied Sciences, Inndalsveien 28, Box 5063, N-5009 Bergen, Norway; Said.Bentouba@hvl.no; 2 Center for Materials Science and Nanotechnology, Department of Chemistry, University of Oslo, Box 1033 Blindern, N-0315 Oslo, Norway; ponniah.vajeeston@smn.uio.no; 3 Department of Chemistry, Faculty of Science, University of Jaffna, Sir. Pon, Ramanathan Rd, Jaffna 40000, Sri Lanka; srtharsha12@gmail.com

**Keywords:** TiO_2_, water-splitting, theoretical, experimental, DFT

## Abstract

Hydrogen produced from water using photocatalysts driven by sunlight is a sustainable way to overcome the intermittency issues of solar power and provide a green alternative to fossil fuels. TiO_2_ has been used as a photocatalyst since the 1970s due to its low cost, earth abundance, and stability. There has been a wide range of research activities in order to enhance the use of TiO_2_ as a photocatalyst using dopants, modifying the surface, or depositing noble metals. However, the issues such as wide bandgap, high electron-hole recombination time, and a large overpotential for the hydrogen evolution reaction (HER) persist as a challenge. Here, we review state-of-the-art experimental and theoretical research on TiO_2_ based photocatalysts and identify challenges that have to be focused on to drive the field further. We conclude with a discussion of four challenges for TiO_2_ photocatalysts—non-standardized presentation of results, bandgap in the ultraviolet (UV) region, lack of collaboration between experimental and theoretical work, and lack of large/small scale production facilities. We also highlight the importance of combining computational modeling with experimental work to make further advances in this exciting field.

## 1. Introduction

Over the last years, there has been a steadily increasing focus on clean, renewable energy sources as a priority to hinder the irreversible climate change the world is facing and to meet the continuously growing energy demand [1]. One hour of solar energy can satisfy the energy consumption of the whole world for a year [2]. Hence, direct harvesting of solar light and its conversion into electrical energy with photovoltaic cells or chemical energy by photoelectrochemical reactions are the most relevant technologies to overcome this challenge. Conventionally, both technologies rely on the collection of light in semiconductor materials with appropriate bandgaps matching the solar spectrum, and thus providing a high-energy conversion efficiency.

Unfortunately, the technology has drawbacks, which prevent it from overtaking non-renewable energy as the main energy source. A major issue is the uneven power distribution caused by varying solar radiation and a lack of proper storage alternatives. As a solution to this problem, the focus is moving toward research on storage options for the produced electricity, which we can divide into mechanical and electrochemical storage systems. For example, in Oceania, pumped hydroelectricity (mechanical) is the most common storage system for excess electricity [3]. Different batteries (lithium–ion, sodium–sulfur (S), vanadium, etc.), hydrogen fuel cells, and supercapacitors are the current focus areas for electrochemical storage [3]. There are several reasons for choosing hydrogen as a way to store solar energy, namely, (1) there is a high abundance of hydrogen from renewable sources; (2) it is eco-friendly when used; (3) hydrogen has a high-energy yield, and (4) it is easy to store as either a gas or a liquid [4,5,6]. 

The high energy yield and ease of storage make hydrogen viable as fuel for the long transport sector; airplanes, cruise ships, trailers, and cargo ships [7,8]. The realization of a green energy shipping fleet could alone yearly cut 2.5% of global greenhouse emissions (GHG) [9]. However, to succeed in this strategy, hydrogen must be produced in a clean and renewable way.

**Figure 1:**
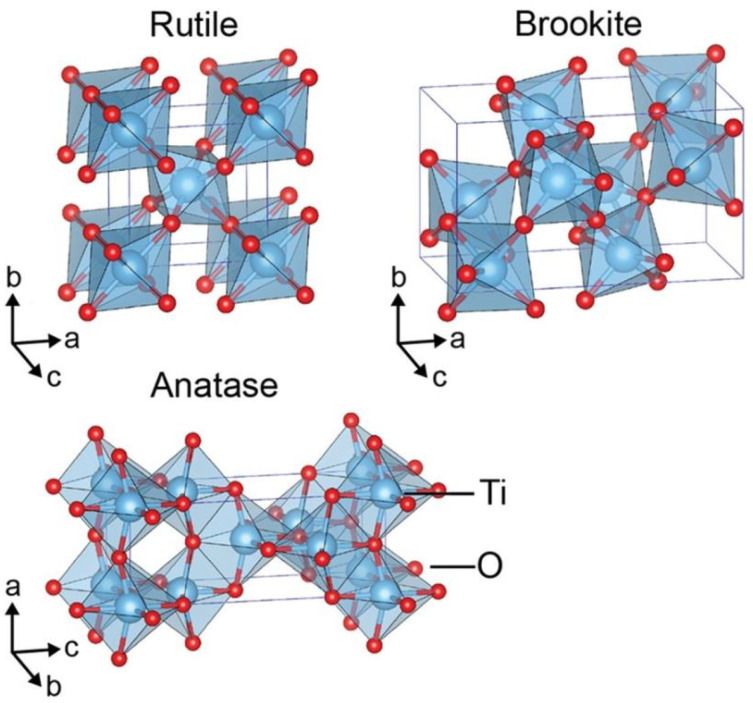
Crystal structures of TiO_2_ rutile (tetragonal, P42/mmm), brookite (orthorhombic, Pbca), and anatase (tetragonal, I41/amd) polymorphs. Reused with permission from [13].

**Figure 2:**
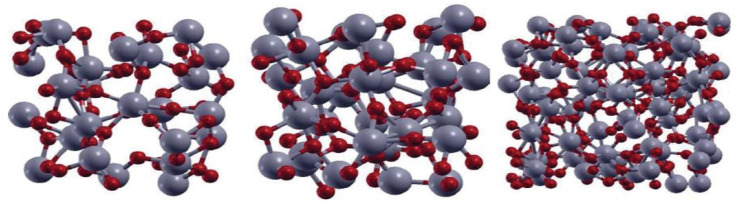
The structure of 72-atom (**left**), 96-atom (**middle**), and 216-atoms (**right**) models of amorphous TiO_2_. The red and grey spheres represent O and Ti atoms respectively. Reused with permission from [14]. Copyright 2012, with permission from Elsevier.

As water splitting got the attention of the researchers in the 1970s, titanium dioxide became the most prominent photocatalyst used [10]. There are several good reasons for this: low cost, chemical stability, earth abundance, and nontoxicity [11]. However, TiO_2_ also sports a wide bandgap (3.0–3.2 eV), which reduces the potential for absorption of visible light [11]. Due to TiO_2_s structural and chemical properties, it is possible to engineer the bandgap, light absorption properties, recombination time, etc. by increasing the active sites and improving the electrical conductivity [12]. TiO_2_ exists in several different polymorphs that all behave differently. The most common ones are rutile, brookite, and anatase as shown in Figure 1. Rutile and anatase TiO_2_ are the most used polymorphs for photocatalytic water splitting; nevertheless, some attempts with amorphous TiO_2_ (aTiO_2_) have been made as shown in Figure 2.

Several attempts have been made to introduce dopants to improve the optical absorption of TiO_2_. For example, Zhang et al. found that 12.5% copper (Cu)-doped anatase TiO_2_ showed a broader absorption peak than pure anatase titanium dioxide [15]. Through a theoretical study, they found that Cu introduces an unoccupied impurity continuum band at the top of the valence band, which explains the improved optical absorption. Another theoretical study, conducted by Morgade and Cabeza in 2017, shows that co-doping of TiO_2_ with (Pt, V) and (C, N) narrows the bandgap and favorably modifies the position of the valence and conduction band edges [16]. Our study will provide an insight into current theoretical and computational studies carried out on water splitting using either pure or doped TiO_2_ semiconductors. In addition, we will compare it with the state of art experimental studies conducted within the field. Our aim is to help bridge the gap between theoretical simulations and experimental research. The two approaches complement each other and when combined could support the field moving forward toward the realization of the hydrogen economy. The theoretical study allows testing of the properties of thousands of different materials with different parameters to gain an understanding of how and why certain dopants and material combinations work. However, the computational models are worked out using perturbation theory, which lowers the overall accuracy of the results. At the same time, an experimental study is important to verify the theoretical results and find the best methods to synthesize the materials in practice.

## 2. Solar-Driven Hydrogen Production 

Most of the commercial production of hydrogen stems from four sources: natural gas, coal, oil, and electrolysis. Of these, steam reforming alone stands for 48% of the world’s hydrogen production, while coal contributes 18%, oil 30%, and electrolysis 4% [17]. The first three hydrogen production processes are energy-consuming and use non-renewable energy sources, which is unattractive for environment protection and climate change [18,19]. However, the production of hydrogen by electrolysis requires only water and electrical current. To have green hydrogen, produced friendly to the environment, we propose to use renewable energy sources—wind, hydro, and solar power—to produce the electric current needed for the electrolysis of water. Solar power is ideal due to the high amount of incoming energy. There are several functional methods used in driving the electrolysis process, i.e., thermochemical water splitting [20], photo-biological water splitting [21], and photocatalytic water splitting [22]. Furthermore, photocatalytic water splitting (PWS) is considered the best option, due to the following reasons: (1) PWS has a good solar-hydrogen conversion efficiency, (2) it has a low production cost, (3) oxygen and hydrogen can easily be separated during the PWS process, and (4) hydrogen electrolysis could be used on both small- and large-scale facilities [4,22,23]. 

### 2.1. Photocatalytic Water Splitting (PWS)

**Figure 3:**
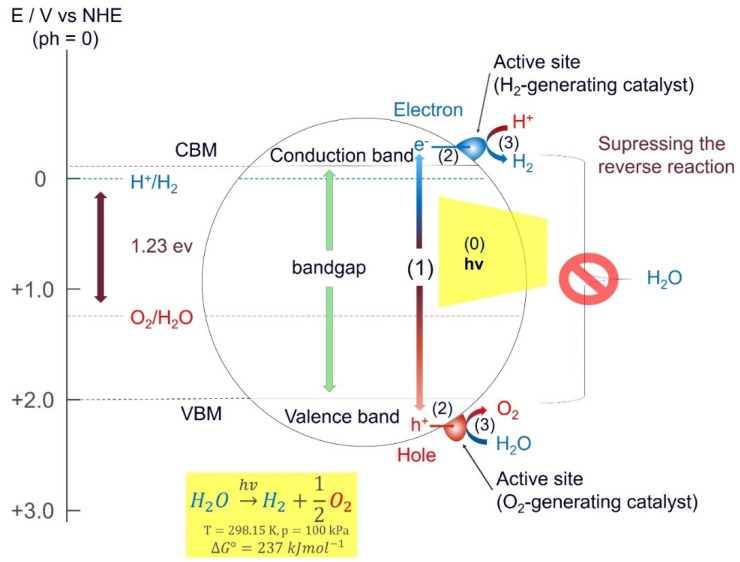
Schematic illustration of the main photocatalytic steps for a semiconductor photocatalyst: (1) light is absorbed to generate electron–hole pairs; (2) migration of excited carriers (electrons and holes) to the surface; (3) surface reaction to produce hydrogen with electrons and oxygen with holes. Reproduced with permission from [25], Copyright 2013, with permission from Elsevier.

The photocatalytic process splits water (H_2_O) into hydrogen (H_2_) and oxygen (O_2_) in the presence of a catalyst and natural light; it is an artificial photosynthesis method. Figure 3 shows a schematic illustration of the major steps involved in the process of photocatalytic water splitting. In the first step (1), electron–hole pairs are generated in the presence of irradiation. This is carried out by utilizing the semiconducting nature of the photocatalyst to excite electrons from the valence band (VB) to the conduction band (CB). Photons with energies larger than the bandgap can excite electrons from the VB to the CB. The second step (2) consists of charge separation and migration of the photogenerated electron-hole pairs. Ideally, all electrons and holes reach the surface without recombination to maximize the efficiency of the photocatalyst. In the final step (3), the electrons, which move from the CB to the surface of the catalyst participate in a reduction reaction and generate hydrogen, and the holes diffuse from the VB to the surface of the photocatalyst involved in an oxidation reaction to form oxygen. In general, the efficiency of the catalyst can be enhanced by including dopants or co-catalysts that include metals or metal oxides, such as Pt, NiO, and RuO_2_, which can act as the active sites via enhancing electron mobility. The redox and oxidations reactions on the surface of the photocatalyst are described by the following equations [24]:

(1) $$\begin{array}{c} \mathrm{Oxidation}:\;H_{2}O+2h^{+}\to 2H^{+}+\frac{1}{2}O_{2}. \end{array}$$

(2) $$\begin{array}{c} \mathrm{Reduction}:\;2H^{+}+2e^{-}\to H_{2}. \end{array}$$

(3) $$\begin{array}{c} \mathrm{Overall}\;\mathrm{reaction}:\;H_{2}O+1.23\;eV\to H_{2}+\frac{1}{2}O_{2}. \end{array}$$

The process of water splitting is highly endothermic and requires a Gibbs free energy of 1.23 eV per electron, which corresponds to light with a wavelength of 1008 nm. This means that the photocatalyst must have a bandgap > 1.23 eV, or else the electrons will not have enough energy to start the reaction. In practice, this limit should be 1.6 eV to 1.8 eV due to some overpotentials [24]. Naturally, it should not be too high either, as that would reduce the amount of visible light the photocatalyst can absorb. This means that it is important to find suitable catalysts with a bandgap between 1.6–2.2 eV to ensure maximum absorption of the incoming light. Another important factor regarding the efficiency of a photocatalyst is the recombination time, i.e., the time it takes for an electron to recombine with a hole. If recombination occurs before the electrons can reach the surface and interact with the water molecules, the energy gets wasted and no redox reaction takes place. Unfortunately, there are only a few materials with sufficient recombination time and a satisfactory bandgap that have been identified. However, a recent study by Takata et. al. demonstrates that it is possible to achieve water splitting without any charge recombination losses [26]. With SrTiO_3_ as the photocatalyst loaded with Rh, Cr, and Co as cocatalysts, they achieved an external quantum efficiency up to 96% at wavelengths between 350 nm and 360 nm [26]. This is equivalent to having an internal quantum efficiency of almost unity. The requirements for having an efficient photocatalyst can be summarized in the solar–hydrogen conversion efficiency (STH) equation [27] as follows:

(4) $$\begin{array}{c} \eta_{\mathrm{STH}}=\eta_{A}\times \eta_{\mathrm{CS}}\times \eta_{\mathrm{CT}}\times \eta_{\mathrm{CR}}.\; \end{array}$$

The STH conversion efficiency depends on (1) the efficiencies of light absorption (η_A_), (2) charge separation (η_CS_), (3) charge transport (η_CT_), and (4) charge collection/reaction efficiency (η_CR_). The efficiency of the photocatalyst depends on several factors and they are elaborated in the following section.

### 2.2. Important Aspects of Photocatalytic Efficiency for Nanomaterials

There are several ways to improve and modify the fundamental properties of a photocatalyst by focusing on its shape, size, order, uniformity, and morphology.

#### 2.2.1. Crystallinity

Research has shown that the crystallinity of the material affects its optoelectronic properties [28,29]. Structures with a high crystallinity perform better than amorphous variations of the same material. The increase in crystallinity reduces the number of defects in the structures and thus decreases the electron-hole recombination sites, which leads to a better catalytic activity [30,31,32,33]. Liu et al. studied the effect of crystalline TiO_2_ nanotubes against that of amorphous TiO_2_ nanotubes and found that better photocurrent properties were attained with the crystalline structures due to the lower amount of electron-hole recombination [34]. In another study, enhanced hydrogen production was obtained using extremely ordered nanotubular TiO_2_ arrays [35].

#### 2.2.2. Dimensionality

Nanomaterials can be classified into four different categories depending on their dimensionality—zero-dimensional (0D), one-dimensional (1D), two-dimensional (2D), and three-dimensional (3D) [36,37]. Zero-dimensional (0D) nanostructures used in PWS are primarily quantum dots (QDs) and hollow shells. In general, QDs are used to decorate the photocatalyst because they increase the visible light absorption and reduce the electron-hole recombination [38,39,40]. One-dimensional (1D) structures include nanorods, nanotubes, and nanowires, which are all attractive for photocatalysts. It is found that nanorod and nanowire arrays result in a more efficient photogenerated electron transport and collection [41,42,43]. On the other hand, nanotubes have a higher surface area for redox reactions compared to nanorods or nanowires although they have less material for light absorption [44,45]. Two-dimensional (2D) nanostructures have a high surface area and a small thickness that reduces the travel distance for generated holes. This results in efficient light harvesting. Lastly, 3D nanostructures are promising candidates for PWS because they can be designed into high-performance photoanodes [27]. In general, it is possible to design and create nanostructures that cater to specific tasks.

#### 2.2.3. Temperature and Pressure

Temperature and pressure during the production phase will affect the resulting properties of a photocatalyst. Research shows that by varying the pressure, the STH performance of the catalyst will change [46]. Another research group found that by using a low-temperature thermal treatment process the charge transfer resistance could be reduced [47].

#### 2.2.4. Size

As mentioned TiO_2_ exist in three phases, anatase (tetragonal; a = 3.7845 Å; c = 9.5143 Å), rutile (tetragonal; a = 4.5937 Å; c = 2.9587 Å), and brookite (orthorhombic; a = 5.4558 Å; b = 9.1819 Å; c = 5.1429 Å). Among the three different crystalline phases of TiO_2_, anatase exhibits the highest stability for particle size less than 11 nm, whereas rutile shows thermodynamic stability for particle size greater than 35 nm, and brookite is stable in the size range of 11–35 nm. The size of the nanomaterials and cocatalyst can alter the overall performance of the system. Smaller particles are dominated by electrokinetics and are thus more suited for photocatalysis. Alternatively, larger particles are better suited for photoelectrochemical (PEC) water splitting because they have a lower electron–hole recombination rate [48]. The size of the particles also influences the electron-hole recombination time. In larger particles the travel distance to the active sites on the surface becomes longer, thus increasing the probability for electron-hole recombination. This probability is decreased in smaller particles due to the shorter migration distance [49,50]. 

#### 2.2.5. Bandgap

The bandgap is one of the most important properties of the photocatalyst. It is defined as the energy needed for an electron to move from the valence band maximum (VBM) to the conduction band minimum (CBM) in a semiconductor. In addition to a fitting bandgap, the CBM must be more negative than the redox potential of H^+^/H_2_ (0 V vs. normal hydrogen electrode (NHE)), while the VBM must be more positive than the redox potential of O_2_/H_2_O (1.23 V). Therefore, the theoretical minimum bandgap for water splitting is 1.23 eV. Nanomaterials are used to tune the band positions and the bandgap toward the appropriate range of 1.6 eV to 2.2 eV [51,52,53].

#### 2.2.6. pH Dependency

The pH value of the solution in which the photocatalyst is placed affects the end STH efficiency [54]. It will similarly affect the stability and lifetime of the catalyst. Photoelectrochemical water splitting is very dependent on the pH of the electrolyte solution, which determines the net total charge adsorbed at the surface of the catalyst. The migration of ions during the reactions may weaken the surface of the electrode. The electrode incorporated with nanomaterials exhibits better stability in different pH conditions, however, it was evident that the stability was further improved when the solution is buffered [55,56,57].

#### 2.2.7. Light

It is important that the light source be specified, as semiconductors doped with nanomaterials can absorb both infrared and UV light in addition to visible light [58].

### 2.3. Theoretical Methods

Numerical studies of electronic, optical and mechanical characterization of TiO_2_ polymorphs are performed as ab initio calculations within the framework of density functional theory (DFT). However, the calculation model and details vary between the researchers depending on the calculation tool/code chosen; for example, Vienna ab initio simulation package (VASP) [59,60,61,62,63,64], CASTEP [65], CRYSTAL [66,67], and GPAW [68]. In general, the interaction between the core and the valence electrons is described by the projector augmented-wave method [69,70]. The electron properties are calculated by G_0_W_0_ [71,72], HSE06 [73,74], or by the generalized gradient approximation (GGA, which is less accurate but faster) [75]. It is possible to calculate the stress tensor by applying a set of strains to the crystal, which leads to the elastic constants (e.g., VASPKIT [76]). Moreover, it is possible to calculate the real space force constants of the supercell, and then to evaluate this with appropriate software (e.g., Phonopy [77]) in order to find the phonon frequencies for dynamical stability, heat capacity, free energy, and entropy analysis. 

### 2.4. Experimental Methods

Several synthesis methods are used for the synthesis of TiO_2_ materials depending on the end application and experiment performed. The methods can roughly be divided into thermal reactions, deposition methods, sol–gel, Micelle, and electromagnetic methods. In general, the thermal methods are heterogeneous reactions in the presence of aqueous solvents or mineralizers under high pressure and temperature [78,79,80]. Deposition methods (e.g., chemical vapor deposition and electrodeposition) are primarily used to create thin-film materials or coatings on a substrate [81,82,83]. Sol–gel methods utilize the conversion of a liquid solution (sol) into a solid gel phase, in which the nanoparticles are formed by hydrolysis and condensation [78,84]. Micelles are the long-chain molecules made by surfactants, which contain a hydrophilic head and a hydrophobic chain. Self-assembling of these amphiphilic molecules forms an organized structure in solution [85]. The ultrasound technique can be used for preparations of a wide range of nanomaterials, especially high-surface-area transition metals, carbides, oxides, alloys, and colloids [84,86]. However, if the goal is to create a dielectric material, then the microwave method can be used [78,87].

## 3. Theoretical Research

The amount of theoretical and computational research has increased over the last years due to improved accuracy of the models and increased computing power. As with experimental work, most of the focus is on various dopants for TiO_2_. The majority of research is conducted on anatase because that phase seems most promising for water splitting. However, rutile TiO_2_ does have some interesting properties.

### 3.1. Metal Dopants

**Figure 4:**
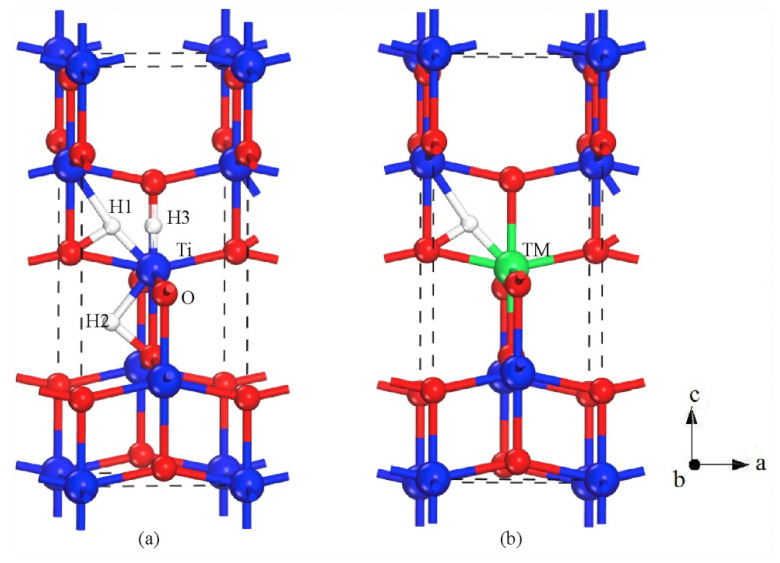
Structural model for (**a**) possible locations of H dopants and (**b**) hydrogenated noble-metal doped TiO_2_. Reprinted with permission from [88]. Copyright 2018, with permission from Elsevier.

**Figure 5:**
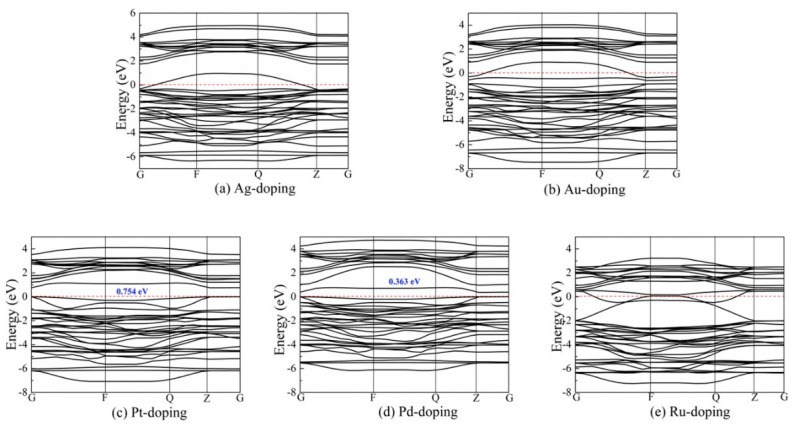
Calculated band structure, using generalized gradient approximation (GGA) with Perdew-Burke-Ernzerhof (PBE) exchange-energy, in the Brillouin zone for, (**a**) Ag-doping, (**b**) Au-doping, (**c**) Pt-doping, (**d**) Pd-doping, and (**e**) Ru-doping. Reprinted with permission from [88]. Copyright 2018, with permission from Elsevier.

A large part of the conducted research is devoted to metal dopants and their contribution to a broadened bandgap of photocatalyst TiO_2_. In a comprehensive study, Pan et al. investigated how noble metals could enhance the catalytic activity of anatase TiO_2_ for hydrogen evolution reaction [88]. They proposed three different structural models for hydrogenated anatase TiO_2_, as seen in Figure 4, and proposed the preferred location (H1 in Figure 4a) of the hydrogen atom when TiO_2_ was noble metal doped. This is because of the strongly localized hybridization between hydrogen and TiO_2_ [88]. Based on these findings, they showed that anatase TiO_2_ was easy to hydrogenate, and the introduced hydrogen could improve the electronic transport between the conduction band and valence band near the Fermi level [88]. In general, silver (Ag)- and gold (Au)-doping are more thermodynamically stable than that of platinum (Pt)-, palladium (Pd)- and ruthenium (Ru)-doping. The band structures for noble metal-doped TiO_2_ are shown in Figure 5, and it is clearly seen that the introduction of dopants reduces the bandgap of TiO_2_. However, Ag-doping seems to be the best option for noble metal doping of TiO_2_ as the other dopants reduce the bandgap below 1.23 eV [88].

Y. Zhang et al. doped (001) anatase TiO_2_ with Pt, cobalt (Co), and Ru [89], which led to surface-localized states that enhanced the electron transfer at the surface. However, another study by S.T Zhang et al. showed that to achieve a stable interface between supported Ru_n_ (*n* = 1–10, 20, 22) clusters and TiO_2_, *n* > 6 was needed [90]. Interestingly enough, this is among the preferred geometries for TiO_2_ [90]. 

Metal dopants decrease the bandgap, and as shown by Jin et al., Pt, Pd, rodhium (Rh), and Ru single atom doping significantly reduces the work function of the compound [91].

Another option to increase the optical absorption is to use iron (Fe) or nickel (Ni) as dopants because they will induce impurity states in the forbidden region [92]. Electrons with energy less than the bandgap can use these impurity states as steps when moving from the valence band to the conduction band. Especially co-doping of TiO_2_ with Fe and Ni results in higher absorption and reduced electron–hole recombination according to Lin et al. [92]. Ghuman et al. also showed that Fe^2+^ doped aTiO_2_ adsorbs water better than pristine aTiO_2_ and that it also has a better photocatalytic effect [93]. 

Introducing Cu and/or N dopants will also create isolated states in the bandgap, and therefore, TiO_2_ doped with these dopants function better than pure anatase TiO_2_ [94]. Assadi et al. showed that the improved photocatalytic activity in Cu/TiO_2_ was because of effective bandgap narrowing and increased charge transfer (electronic interactions) and not surface chemistry [95]. Wei Zhang et al. found that the stability of Cu doped TiO_2_ depends on which oxygen atoms that is replaced with Cu atoms. [15] They observed a blueshift in absorption for anatase TiO_2_ (101) compared to bulk TiO_2_, while in Cu-doped bulk anatase TiO_2_ they observed a redshift in optical absorption [15]. Co-doped SrTiO_3_ has a narrower bandgap compared to that of pure TiO_2_ according to Sikam et al. and this is due to states being formed in the gap [96]. In addition, they also found that co-doping resulted in magnetism due to inequality of spin down and spin up states [96]. Ghuman et al. looked into the difference between monodoping and co-doping using nitrogen (N) and niobium (Nb) on amorphous TiO_2_ [97]. They found that monodoping reduces the bandgap but it also increases the number of recombination centers [97]. Charge compensated co-doping, on the other hand, reduces the bandgap with 0.4 eV and suppresses the recombination effect by eliminating band gap states [97]. S and Nb co-doping of anatase TiO_2_ resulted in a bandgap of 2.15 eV as shown by Ren et al. [98].

However, not all states in the bandgap are appreciated; Gao et al. looked into how Mg doping could reduce the shallow defect states under the CBM in TiO_2_, increasing the photocatalytic effect [99].

Although several different dopants have been proposed and tested, not all of them are viable to be incorporated into TiO_2_. Chen et al. used cerium (Ce), praseodymium (Pr), europium (Eu), and gadolinium (Gd) dopants to see how they would incorporate with TiO_2_ [100]. They found that Ce was the easiest among these, while Pr and Gd had low substitutional energy and should be able to be incorporated into TiO_2_ [100]. Eu on the other hand was difficult to incorporate with TiO_2_ [100]. Ce, Pr, and Eu monodoping should move the light absorption more toward/into the visible light region [100]. Another approach when using lanthanides could be ion triple doping as showcased by Li et al. [101]. Through co- and triple-doping they improved the oxidizing ability, light absorption, and charge carrier separation of their photocatalyst (Bi_2_MoO_6_) [101].

### 3.2. Non-Metal Dopants

**Figure 6:**
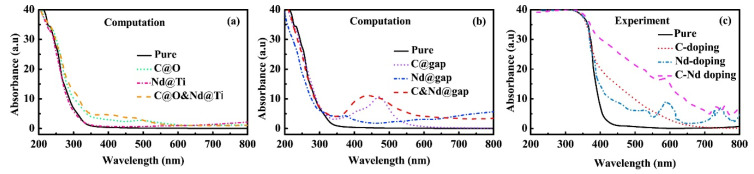
Theoretical and experimental optical absorption spectra for pure and doped TiO_2_ structures. (**a**) Computational obtained spectra for substitutional doping, (**b**) computational obtained spectra for TiO_2_ doped with the dopants at different interstitial sites, and (**c**) experimental obtained optical absorption spectra for C, Nd, and C+Nd doped TiO_2_ from [107]. Reprinted with permission from [102]. Copyright 2017, with permission from Elsevier.

In an attempt to find non-metal dopant alternatives Shi et al. co-doped anatase TiO_2_ with C and neodymium (Nd) and found tuned band gaps, which were lower than that of pure TiO_2_: C@O and Nd@Ti, 2.372 Ev, and carbon (C) and Nd @ TiO_2_, 2.850 eV [102]. The main function of the C and Nd dopants is to enhance the intensity of light absorption and to extend the optical absorption range into the visible light region respectively. This is clearly seen from Figure 6, in which the optical absorption spectra of TiO_2_ do not extend into the visible light region, while both C doping and C and Nd doping extend the absorption range into the visible light region and effectively enhancing the efficiency of the photocatalysts. Experimental studies shown in Figure 6c confirmed these findings. By using graphite carbon spheres on TiO_2_ Jiang et al. introduced isolated energy levels between the VBM and the CBM [103]. These intermediate bandgaps increase the number of electrons that could be excited from the VB to the CB.

Gurkan et al. found that selenium (Se) doping introduces localized mid-gap levels that increase the visible light photocatalytic effect [104]. However, no significant change in the position of the band edges was observed. A study by Zhao et al. showed that N, Co, Co–N, Co–2N, and Co–3N dopants all increased the optical absorption rate compared to that of undoped TiO_2_ [105]. Unfortunately, Co–N and Co–3N shift the CBM below the H^+^/H_2_ reduction potential, which means that it is not useful for water splitting [105]. Pengfei Wang et al. revealed that carbonate could be incorporated into mesoporous TiO_2_ and significantly improve the visible light hydrogen evolution [106]. In addition, the intimate homo-junctions between anatase and rutile phases and graphite carbon on the surface of TiO_2_ can significantly help promote the separation of charge carriers [106].

In an attempt to decrease the bandgap of TiO_2_, Zongyan et al. used boron (B), C, N, fluorine (F), phosphorus (P), S, and chlorine (Cl) as dopants on anatase TiO_2_ [108]. The tuned bandgaps for TiO_2_ with the dopants (B, C, N, P and S) were 2.72 eV, 2.44 eV, 2.74 eV, 2.38 eV, and 2.59 eV, respectively [108]. The improvements are due to new impurity energy levels, which red-shift their fundamental absorption edges to the visible light region [108]. In addition, they found that higher dipole moments could lead to easier separation of the photoexcited electron-hole pairs, which would increase the photocatalytic effect [108].

There has been some research on TiO_2_/g–C_3_N_4_ heterostructure photocatalysts. For example, Yali Zhao et al. co-doped TiO_2_/g–C_3_N_4_ with Cu and N [109]. This resulted in an obvious narrowing of the bandgap compared with pure TiO_2_, co-doping induces impurity states of N 2p and hybridized states of Cu 3d and N 2p in the bandgap of TiO_2_/g–C_3_N_4_ [109]. Yanming Lin et al. used TiO_2_/g–C_3_N_4_ heterostructure through interfacial coupling for H_2_ production. The calculated band gap was significantly reduced compared to pure TiO_2_ [110]. TiO_2_/g–C_3_N_4_ heterostructure also has a higher CBM energy, which provides the photoexcited electrons with stronger reducing power to produce more hydrogen per unit time compared with TiO_2_ [110]. 

### 3.3. Rutile 

Although most researchers focus on anatase TiO_2_, there has been some development on rutile TiO_2_ as well. Atanelov et al. doped rutile TiO_2_ with C and it seems to have worse photocatalytic performances than pure TiO_2_, this is due to C–O dimes creating mid-band states [111]. However, N doped rutile TiO_2_ introduced no mid-bandgap states [111]. Ghuman et al. used Rh to dope rutile TiO_2_ and found that it had a lower bandgap but with more recombination centers [112]. By using Rh–Nb (charge compensated doping) as co-dopants on rutile TiO_2_, they obtained no isolated bandgap states that might act as a recombination center [112]. Moreover, the bandgap was decreased by 0.5 eV, which makes it a better photocatalyst [112].

### 3.4. Nanotubes

**Figure 7:**
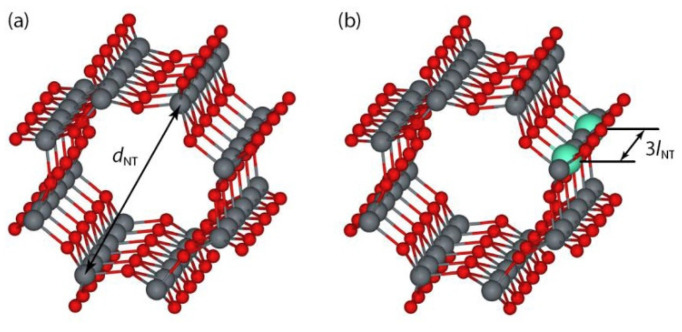
Structural models of non-optimized (**a**) pristine and (**b**) doped armchair type (4,4) fluorite-structured TiO_2_ nanotubes. The red balls represent O, grey balls are TiO_2_, and the turquoise balls represent the 3d-metal dopants substituted for Ti atoms. The nanotube diameter (d_nt_) is 0.84 nm and the dopant concentration is considered to be 4.17%. Reprinted with permission from [115]. Copyright 2017, with permission from Elsevier.

Research on different TiO_2_ nanostructures has led to some interesting results. Lisovski et al. doped TiO_2_ nanotubes with sulfur and achieved a narrower bandgap compared to that of pure TiO_2_ [113]. The band edges were also close to the limits for efficient water splitting [113]. In another article, Lisovski et al. present nitrogen (N), S, and S-and-N doping of six-layer (101) anatase TiO_2_ nanotubes [114]. They found that monodoping with N or S reduces the photocatalytic effect of the nanotubes [114]. However, co-doping with N and S could improve the photocatalytic activity, although it depends on the defect concentration [114]. Dmitry Bocharov et al. used TiO_2_ (4,4) nanotubes doped with scandium (Sc), depicted in Figure 7, and found that they were a good candidate with a bandgap of 1.8–1.9 eV [115]. Working on the same variation of nanotubes, E.P. D’yachkov et al. found that doping with Nb, molybdenum (Mo), technetium (Tc), and Pd leads to bandgaps around 2 eV [116]. This is a significant decrease from around 4 eV for undoped (4,4) TiO_2_ nanotubes [116]. Lisovski et al. also investigated if the arrangement of bandgap edges would change when going from bulk to nanotubes. They found that this only happened if the diameter of the nanotubes was small, i.e., the internal strain was extremely large [117].

### 3.5. Pure TiO_2_

Naturally, some focus had been on pure TiO_2_ and its different states, looking into which one was most suited for photocatalytic activity. 

Ma et al. found that the (101) facet had higher activity compared to other facets of TiO_2_ [118], which was an important breakthrough. If we have pristine conditions and water molecules, water splitting can be expected both with rutile and anatase TiO_2_ according to Deak et al. [119]. This is generally not the case because the surface will contain bridging OH^+^ and the terminal OH^−^ (dissociated water). In these conditions, anatase TiO_2_ is the best for water splitting [119]. However, by increasing the OH^+^/OH^−^ ratio, one can increase the driving force for water splitting [119]. Hanaor et al. [120] looked into the phase stability of anatase and rutile TiO_2_, with and without doping. Pure TiO_2_ has a more stable rutile phase than anatase and the transformation from anatase TiO_2_ to rutile TiO_2_ is irreversible [120]. F, Si, Fe, and Al as dopants work as inhibitors for the transformation process, and they slow down the process from anatase to rutile [120]. Alghamdi et al. report no sign of overlapping HOMO levels between H_2_O_2_ and TiO_2_ rutile (110) surface; this in addition to the high adsorption energy could explain why water splitting is slow [121].

### 3.6. Collected Data

In Table 1 we have tabulated the bandgap, photocurrent density, hydrogen, and oxygen production rate from the articles discussed in this section.

As Table 1 clearly illustrates, the major focus for theoretical studies had been on bandgap calculations and alterations, but these numerical results had deviations from the results from experimental research. The majority of dopants introduced intermediate bandgaps to the structure, thus reducing the needed phonon energy for excitation. We find that by choosing the correct dopant, the effective bandgap can be lowered to 2.15 eV by co-doping TiO_2_ with sulfur and niobium [98]. Other promising candidates are N-doped aTiO_2_ (2.25 eV) [97] and the more complex g-C_3_N_4_/TiO_2_ (2.21 eV) [110]. A general problem with the computational studies we have reviewed is the lack of a successful descriptor of the hydrogen evolution reaction (HER) activity, however, the hydrogen adsorption free energy, ΔG_H_, has shown promises [122]. 

## 4. Experimental Research

Recent advances in fabrication techniques have made it possible to deposit ultra-thin films, various-sized nanoparticles, and to create nanowires, nanorods, nanobelts, etc. This has made it possible to utilize interesting properties of nanostructure and improve TiO_2_ photocatalysts. 

### 4.1. Metal Dopants 

An interesting phenomenon that could be exploited to increase the solar absorption is the surface plasmon resonance (SPR) effect and localized surface plasmon resonance (LSPR) effect, where metal nanoparticles absorb incoming light outside the bandgap of the catalyst. The generated electrons will then be transferred to the surface of the photocatalyst and take part in the oxidation and reduction of the water molecules. To achieve this, one must add metal nanoparticles to the surface of the photocatalyst and let them absorb incoming radiation. Several attempts utilizing metal dopants on TiO_2_ photocatalysts have revealed an increase in light absorption due to SPR/LSPR. 

Zhao Li et al. worked on aluminum-doped TiO_2_, and they report an increased PEC efficiency due to the LSPR effect [123]. Interestingly enough, they also found that an ultrathin (0.8–2.5 nm) layer of Al_2_O_3_ is formed naturally, which works as a protective layer against Al NPs corrosion and in reducing the surface charge recombination [123]. 

A similar increase in light absorption due to SPR and LSPR can be found using Co, Ni, titanium nitride (TiN), Au, Cu, or Ag dopants [124,125,126,127,128,129]. Nickel was also found to improve the separation of electron–hole pairs [125], while TiN assisted with charge generation, separation transportation, and injection efficiency [126]. Another advantage of the Ag nanoparticles is that they lower the charge carrier recombination rate [129]. In their research on Au dopants, Jinse Park et al. used ZnO–TiO_2_ nanowires and found that the nanowires themselves excel in charge separation and transportation [127]. Shuai Zhang et al. used a Cu doped TiO_2_ nanowire film, which showcased clear improvement in photocurrent density due to the unique architecture [128]. 

In a similar experiment, Jie Liu et al. used Co_3_O_4_ quantum dots on TiO_2_ nanobelts and achieved H_2_ and O_2_ production rates of 41.8 and 22.0 µmol/hg [130]. The QDs favored transfer and accommodation of photo-generated electrons, in addition, to inhibit the recombination of charge carriers [130]. 

Doping could also induce a Schottky junction in the photocatalyst, which could help increase the charge transfer and help separate the photogenerated electrons and holes. He et al. showed this, using TiO_2_ nanowire decorated with Pd NPs and achieving a photocurrent density of 1.4 mA/cm^2^ [131]. The use of platinum within TiO_2_ based photocatalysts is well known, and Lichao Wang et al. showed that by creating a Pt/TiO_2_ photocatalyst, an H_2_ production rate of 7410 µmol/gh is achievable [132].

Complex dopants have also been used on TiO_2_, in addition to nanostructures. This makes it possible to combine the properties of the various dopants on TiO_2_. In an attempt to increase the hydrogen production of TiO_2_, Ejaz Hussain et al. doped TiO_2_ with Pd–BaO NPs [133]. They achieved an H_2_ production of 29.6 mmol/hg in a solution of 5% ethanol and 95% water [133]. Hussain et al. took advantage of the inherent high catalytic activity of the Pd nanoparticles, and the fact that barium oxide (BaO) enhances the electron transfer from the semiconductor band to the Pd centers [133]. In a similar approach, cadmium sulfide (CdS) was incorporated into a TiO_2_ photoanode [134]. This utilized the suppression of electron-hole recombination and efficient charge separation/diffusion due to the nanorod structure, in addition to the SPR effect from the dopants [134]. Instead of doping TiO_2_ only with CdS, it could be combined with tin (IV) oxide (SnO_2_) nanosheets. The reason for this is that TiO_2_ reduces the charge recombination between Cds and SnO_2_ [135]. Thus, the number of electrons and holes reaching the surface and participating in the reduction or oxidation process increases. 

It is also possible to dope TiO_2_ with Ti^3+^ and Ni to improve the overall efficiency; in fact, Ti^3+^/Ni co-doped TiO_2_ nanotubes have a bandgap of 2.84 eV [136]. This is roughly 12% narrower than that of pure TiO_2_ and could be explained by the SPR effect. 

Lately, there has been some research devoted to black titanium dioxide. Mengqiao Hu et al. used Ti^3+^ self-doped mesoporous black TiO_2_/SiO_2_/g–C_3_N_4_ sheets [137]. The system has a bandgap of ~2.25 eV and photocatalytic hydrogen evolution of 572.6 µmol/gh. This is all due to the unique mesoporous framework enhancing the adsorption of pollutants and favoring the mass transfer, Ti^3+^ self-doping reducing the bandgap, and extending the photoresponse to the visible light region [137]. 

**Figure 8:**
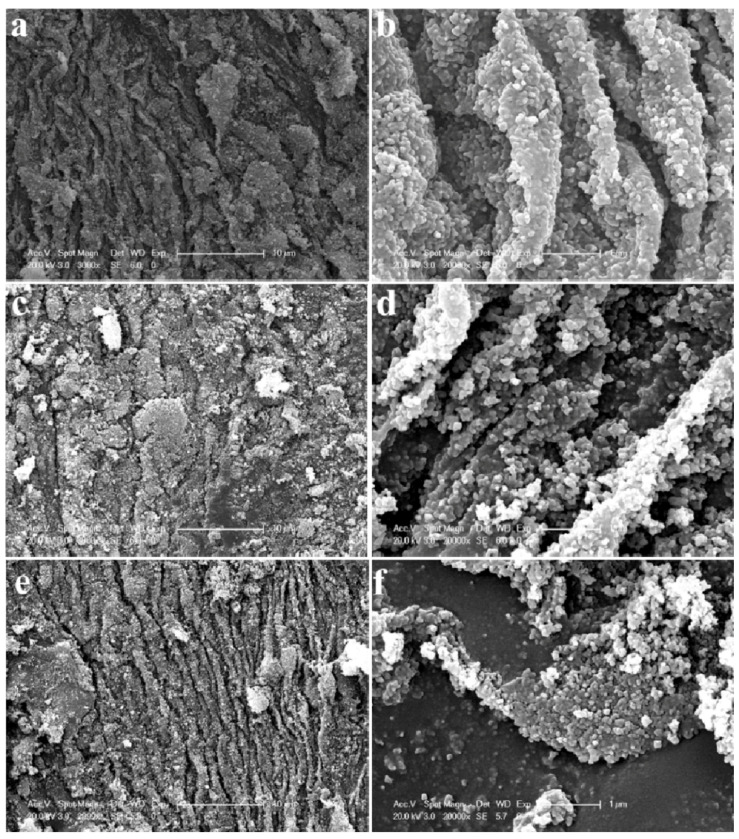
SEM images of MoSe_2_ with TiO_2_ nanoparticles synthesised using a simple hydrothermal method; (**a,b**) 0.025%, (**c,d**) 0,05%, and (**e,f**) 0.1% mass ratio of MoSe_2_:TiO_2_. Reprinted with permission from [138]. Copyright 2019, with permission from Elsevier.

Through modifying the TiO_2_ NPs with 2D molybdenum disulfide (MoSe_2_), Lulu Wu et al. achieved a hydrogen production rate of 5.13 µmol/h for samples with 0.1 wt.% MoSe2 [138]. They created MoSe_2_ nanosheets, which were then combined with TiO_2_ nanoparticles to create an efficient photocatalyst (Figure 8), by taking advantage of the wide light response and rapid charge migration ability of 2D nanosheets MoSe_2_. A slightly different approach would be to wrap rutile TiO_2_ nanorods with amorphous Ta_2_O_x_N_y_ to achieve an optical bandgap of 2.86 eV with band edge positions suitable for water splitting [139]. 

Bismuth vanadate (BiVO_4_), iron (III) oxide/hematite (Fe_2_O_3_), and bismuth ferrite (BiFeO_3_) are materials with interesting properties for solar-driven water splitting. They all have low bandgaps, which could help with visible light absorption, and are both simple and inexpensive materials [140,141,142] 

Xin Wu et al. utilized BiFeO_3_ (BFO) on top of TiO_2_ and found a photocurrent density as high as 11.25 mA/cm^2^, 20 times higher than that of bare TiO_2_ [143]. The improvement is mainly due to the heterostructure of BFO/TiO_2_ and the ferroelectric polarization due to the introduction of BFO, which could lead to upward bending at the interface and thus effectively drive the separation and transportation of photogenerated carriers [143].

Bismuth vanadate is most often used together with a dopant. For example, Jia et al. used W to dope TiO_2_/BiVO_4_ nanorods and obtained a bandgap of 2.4 eV [144]. In addition, Wengfeng Zhou et al. synthesized an ultrathin Ti/TiO_2_/BiVO_4_ nanosheet heterojunction [145]. It had an enhanced photocatalytic effect due to the formation of a built-in electric field in the heterojunction between TiO_2_ and BiVO_4_ [145]. Using Co, Pi Quan Liu et al. modified a TiO_2_/BiVO_4_ composite photoanode, which shows improved visible light absorption and a more efficient charge transfer relay [146]. By combining FeOOH/TiO_2_/BiVO_4_, Xiang Yin et al. created a photoanode that led to a hydrogen production rate of 2.36 µmol/cm^2^ after testing for 2.5 h [147].

However, it is possible to use BiVO_4_ without a dopant BiV because O_4_ and TiO_2_ naturally complement each other. It allows for the exploitation of the excellent absorption properties of BiVO_4_ to produce highly reductive electrons through TiO_2_ sensitization under visible light [148]. Another example of this is how Ahmad Radzi et al. deposited BiVO_4_ on TiO_2_ to increase PEC efficiency [149]. 

Hematite is usually combined with more complex structures, for example, 3d ordered urchin-like TiO_2_@Fe_2_O_3_ arrays [150]. Using these arrays Chai et al. reported a photocurrent density of 1.58 mA/cm^2^ at 1.23 V vs. reversible hydrogen electrode (RHE) [150]. This is a clear improvement compared to pristine TiO_2_.

A different approach is to use amorphous Fe_2_O_3_ with TiO_2_ and silicon (Si). With this method, Zhang et al. achieved a photocurrent density of 3.5 mA/cm^2^ at 1.23 V vs. RHE [151]. It is also possible to use TiO_2_ as the dopant on hematite. Fan Feng et al. decorated a hematite PEC with TiO_2_ at the grain boundaries [152] that increased the charge carrier density and improved the charge separation efficiency, resulting in a photocurrent density of 2.90 mA/cm^2^ at 1.23 V vs. RHE [152].

However, one could also use a simple TiO_2_/Fe_2_O_3_ heterojunction, which Deng et al. found improved the photocurrent density due to improved separation and transfer of photogenerated carriers [153].

A few studies are also reported on on metal-organic frameworks (MOFs) in cooperation with TiO_2_.

Yoon et al. coated TiO_2_ nanorods (NRs) with NH_2_–MIL-125(Ti) and achieved a photocurrent density of 1.62 mA/cm^2^ [154]. The high photocurrent can be explained by several factors: the large surface area and crystallinity of TiO_2_ NRs, which leads to effective light absorption and charge transport. Or the moderate bandgap of MIL(125)–NH_2_, the uniform and conformal coating of the MIL layer, and the efficient charge carrier separation and transportation through the type (II) band alignment of TiO_2_ and MIL(125)–NH_2_.

### 4.2. Non-Metal Dopants

Metal dopants could act as recombination centers for electrons and holes and thus lowering the overall efficiency of the photocatalyst [155]. Thus, a large number of research studies have been going on toward doping TiO_2_ with non-metal dopants, for example, Si, S, C, F, and N. 

Yang Lu et al. doped TiO_2_ nanowires with earth-abundant Si and achieved an 18.2 times increase in the charge carrier density, which was better than in N and Ti (III) doped TiO_2_ [156]. The increase in visible light photocatalytic activity is due to the enhanced electron transport, because of higher charge-carrier density, longer electron lifetime, and larger diffusion coefficient in Si-doped TiO_2_ NWs [156]. High-quality graphene could be of use in water splitting as quantum dots on rutile TiO_2_ nanoflowers because they are highly luminescent and can absorb UV and visible light up to wavelengths of 700 nm [157]. Bellamkonda et al. found that multiwalled carbon nanotubes–graphene–TiO_2_ (CNT–GR–TiO_2_) could achieve a hydrogen production rate of 29 mmol/hg (19 mmol/hg for anatase TiO_2_) [158]. They also had an estimated solar energy conversion efficiency of 14.6% and a bandgap of 2.79 eV, which was due to the generation of Ti^3+^ and oxygen vacancies within the composite [158]. TiO_2_ absorbs UV light due to its inherent large bandgap, Qiongzhi Gao et al. [159] utilized this and doped TiO_2_ with hydrogenated F. The hydrogen-treated F atoms increased both UV and visible light absorption. When TiO_2_ is doped with sulfur, an abundant element, a bandgap of 2.15 eV can be expected [160]. N and lanthanum (La) co-doping of TiO_2_ does not reduce the bandgap, but the photocatalytic effect is seen to be enhanced due to an increase in surface nitrogen and oxygen vacancies [161]. 

### 4.3. Improved Production Methods

As discussed, although there are several options in order to dope TiO_2_ with metals and non-metals, the research community has devoted much energy to improving the characteristics of TiO_2_ photocatalysts through appropriate synthesis conditions. It is possible to achieve improved mechanical strength, enhanced composite stability, surface area, roughness, and fill factor for TiO_2_ by using branched multiphase TiO_2_ [162].

Treating TiO_2_ with Ar/NH_3_ during the fabrication process, which could improve the density of the charge carrier and broaden the photon absorption both in the UV and visible light regions [163]. An increase in density of states at the surface and a 2.5-fold increase in photocurrent density at 1.23 vs. RHE could be achieved by anodizing and annealing TiO_2_ during the fabrication process [164]. Ning Wei et al. showed that by controlling TiO_2_ core shells, it was possible to achieve a bandgap of 2.81 eV, and had an H_2_ evolution rate of 49.2 µmol/(h cm) [165].

Huali Huang et al. looked into the effect of annealing the atmosphere on the performance of TiO_2_ NR [46]. Oxygen, air, nitrogen, and argon were used as the different atmospheres. The same rutile phase was observed, but it resulted in different H_2_ activities. Samples annealed in argon showed the highest photocurrent density of 0.978 mA/cm^2^ at 1.23 V vs. RHE [46], an increase of 124.8% compared to the oxygen annealed samples. It was found that the density of oxygen vacancies in the samples increased with the decrease in oxygen in the annealing atmosphere [46]. The increase in oxygen vacancies enhances visible light absorption and increases the electron conductivity (inhibits recombination of the charge carriers) [46].

Aleksander et al. examined what would happen if the substrate, which TiO_2_ was fabricated on was changed [166]. The authors lowered the optical reflection by using black silicon, which in turn increased the light collection [166]. They also found that the addition of noble metals could induce SPR in the visible light region [166]. 

**Figure 9:**
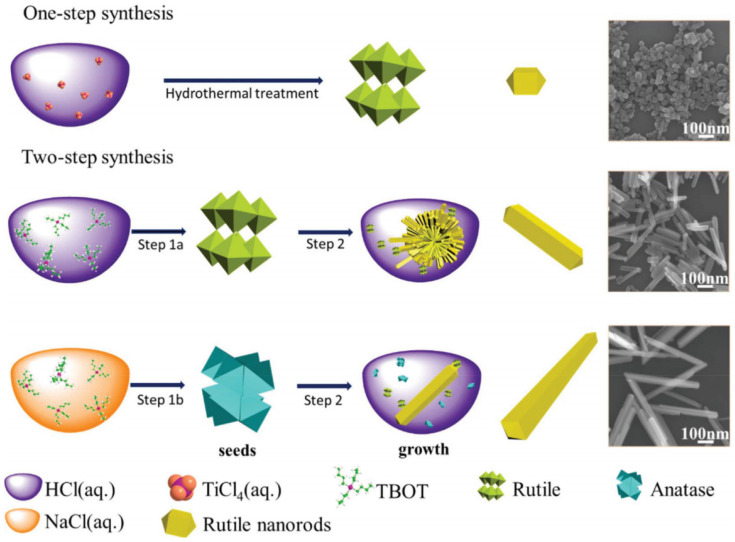
Schematic illustration of the synthesis of rutile TiO_2_ with specific (small, medium, and large) aspect ratios. Reprinted with permission from [167]. Copyright 2018, with permission from RSC.

By combining improved production methods and doping/co-doping with metals/non-metals, TiO_2_ could be realized as an efficient photocatalyst. Fu et. al. showed that by controlling the HCl concentration during the synthesis process, it was possible to synthesize well-crystallized rutile TiO_2_ nanorods with special aspect ratios [167]. They proposed a process, as shown in Figure 9, for synthesizing rutile TiO_2_ with different aspect ratios. Rutile TiO_2_ nanorods with small aspect ratios were formed by placing titanium tetrachloride (TiCl_4_) in liquid hydrochloric acid (HCl) before undertaking hydrothermal treatment. The key factors were the presence of Cl^−^ and H^+^ at high temperature and pressure. For the synthesis of nanorods with medium/large aspect ratios titanium butoxide (TBOT) was added dropwise to HCL (aq.)/NaCl (aq.) This created rutile/anatase crystal seeds, which were placed in HCl (aq.) for the final growth process. They concluded that with decreasing aspect ratios, the photocatalytic water splitting activity would increase for TiO_2_ nanorods [167].

### 4.4. Collected Data

We present in Table 2 the obtained bandgap, photocurrent density, H_2_, and O_2_ production rate values from experimental studies that are reviewed here.

We see from the data presented in Table 2 that it is possible to adjust the properties of TiO_2_ photocatalysts by doping or through structural changes. For example, Elbakkay et al. achieved a bandgap of 2.15 eV using an S–TiO_2_/S-reduced graphene oxide catalyst [160]. Both theoretical and experimental studies point at sulfur as a possible dopant for TiO_2_ that could drastically reduce the effective bandgap of the photocatalyst. In general, theoretical studies tend to focus on simpler structures and doping of TiO_2_, while experimental research has moved on toward more complex structures of TiO_2_ that consist of several layers, materials, and nanostructures. 

It is clear from Table 2 that the various solutions and potentials are used for measuring HER, and presenting photocurrent density in different ways is an issue that makes the direct comparison difficult. This hampers the evaluation of the most promising TiO_2_ structure for photocatalytic activity. Likewise, the theoretical studies use different models and approximations that make it difficult to compare the numerical results for different TiO_2_ structures. Combined theoretical and experimental study along with the establishment of standards, for example, for measuring H_2_ production, would help the path to develop TiO_2_ photocatalysts towards commercial realization. 

**Table 2:** This table displays the bandgap, photocurrent density, and H_2_ production rate experimentally achieved for doped/modified TiO_2_ structures in articles reviewed in this study.

Nanomaterial	Bandgap [eV]	Photocurrent Density at 1.23 V vs. RHE [mA/cm^2^]	H_2_ Production Rate 1.5G Sunlight Bias at 1.23 vs. RHE	O_2_ Production Rate 1.5G Sunlight Bias at 1.23 vs. RHE	Ref.
TiO_2_@Fe_2_O_3_/TiO_2_	2.2	1.58, and 3.6 at 1.6 V vs. RHE	NA	NA	[150]
α-Ta_2_O_x_N_y_ enwrapped TiO_2_ rutile nanorods	2.86	1.32	244.2 mmol/m^2^h	112.7 mmol/m^2^h	[139]
Ag-TiO_2_-NR05	2.64	0.08 and 0.10 mA/cm^2^ at 1.2 and 1.6 V vs. RHE	NA	NA	[129]
W-TiO_2_/BiVO_4_ nanorods	2.4	2.5	41 µmol/h	NA	[144]
Branched multiphase TiO_2_	3.04	0.95	NA	NA	[162]
Co_3_O_4_ quantum dots on TiO_2_	3.07	0.0005	41.8 µmol/h/g	22.0 µmol/h/g	[130]
Co-Pi modified 3D TiO_2_/BiVO_4_	NA	4.96 at 0.63 V vs. Ag/AgCl	NA	NA	[146]
Co doped TiO_2_ nanotubes	2.88	1.0	NA	NA	[124]
Controllable TiO_2_ core shells	2.81	3.88	49.2 µmol/cm^2^h	25.2 µmol/cm^2^h	[165]
A-Fe_2_O_3_/TiO_2_/Si	NA	3.5	NA	NA	[151]
Al@TiO_2_	NA	NA	NA	NA	[123]
Si-doped TiO_2_ nanowires	NA	NA	NA	NA	[156]
Three-layer (SiO_2_, Al_2_O_3_, and TiO_2_) structure with Au particles for LSPR	NA	NA	NA	NA	[168]
BiFeO_3_/TiO_2_	NA	11.25	NA	NA	[143]
Graphene QDs decorated rutile TiO_2_ nanoflowers	NA	~0.32 at 0.5 V vs. Ag/AgCl	NA	NA	[157]
Hierarchical TiO_2_/Fe_2_O_3_	NA	1.79	NA	NA	[153]
CNT-GR-TiO_2_	2.79	NA	29 mmol/h/g	NA	[158]
SnO_2_ nanosheets with TiO_2_ and CdS QD	NA	4.7 at 0V vs. Ag/AgCl	NA	NA	[135]
TiO_2_ nanotubes treated with Ar/NH_3+_	NA	1 at 1.18 V vs. RHE	NA	NA	[163]
TiO_2_ nanowire decorated with Pd	NA	1.4	NA	NA	[131]
NH_2_-MIL-125(Yi) on TiO_2_ nanorods	NA	1.62	NA	NA	[154]
Ni-doped TiO_2_ nanotubes	NA	0.93 at 0 V vs. Ag/AgCl	NA	NA	[125]
N doped La/TiO_2_	2.96–2.99	NA	8.25 µmol/h/g	NA	[161]
TiN boosted N doped TiO_2_	NA	3.12	NA	NA	[126]
CuO@TiO_2_ nanowires	NA	0.56	NA	NA	[128]
Pd-BaO NPs on TiO_2_	NA	NA	29.6 mmol/h/g	NA	[133]
S-TiO_2_/S-RGO	2.15	3.36 at 1 V vs. Ag/AgCl	NA	NA	[160]
Anodized and H_2_ annealed TiO_2_	NA	2.5 fold TiO_2_	NA	NA	[164]
TiO_2_ NPs modified with 2D MoSe_2_	NA	NA	5.12 µmol/h	NA	[138]
Ultrathin Ti/TiO_2_/BiVO_4_	NA	5.8 µa/cm^2^ at 0.5 V vs. Ag/AgCl	NA	NA	[145]
TiO_2_ on black Si	NA	NA	NA	NA	[166]
ZnO-TiO_2_ core-shell nanowires decorated with Au NPs	NA	1.63	NA	NA	[127]
TiO_2_/CdS system	2.25	30 mA/cm^2^ (at 1 V vs. Ag/AgCl) under 1.5 AM	1.3 mmol/cm^2^h	NA	[134]
FeOOH/TiO_2_/BiVO_4_	NA	3.21	2.36 µmol/cm^2^	1.09 µmol/cm^2^h	[147]
hematite PEC decorated with TiO_2_ at the grain boundaries	NA	2.90	NA	NA	[152]
the effect of annealing atmosphere on the performance of TiO_2_ NR	NA	0.978	NA	NA	[46]
Ti^3+^/Ni co-doped TiO_2_ nanotubes	2.84	0.87	NA	NA	[136]
Hydrogenated F-doped TiO_2_	3.0	NA	3.76 mmol/h/g	NA	[159]
BiVO_4_ deposited on TiO_2_	NA	35 µ under 100 mW/cm^2^ in 0.5M Na_2_SO_4_	NA	NA	[149]
BiVO_4_ used together with TiO_2_	NA	~0.3 at 1.0 V vs. RHE	NA	NA	[148]
Pt/TiO_2_(anatase) photocatalyst	NA	NA	7410 µmol/h/g	5096 µmol/h/g	[132]
Ti^3+^ self-doped mesoporous black TiO_2_/SiO_2_/g-C_3_N_4_ sheets	~2.25	NA	NA	NA	[137]
Rutile TiO_2_ nanorods with small aspect ratio	NA	NA	1229 µmol/h/g	549 µmol/h/g	[167]
Rutile TiO_2_ nanorods with medium aspect ratio	NA	NA	783 µmol/h/g	369 µmol/h/g	[167]
Rutile TiO_2_ nanorods with large aspect ratio	NA	NA	549 µmol/h/g	252 µmol/h/g	[167]

As we can observe from the results presented so far, great steps have been taken to make TiO_2_ photocatalysts for water splitting a viable technology for green hydrogen production. However, even though the progress has been rapid over the past decade there are still obstacles in the way to large-scale production facilities.

Coordinated theoretical and experimental study of TiO_2_ structures for enhancing the electronic, optical, and physical properties will help achieve the goal of efficient low-cost photocatalysts for water splitting.

In general, during the synthesis of the photocatalysts, there is uncertainty in the exact composition and structure of the compound [169,170]. This is especially prevalent for doping and the location of the dopants in the compound. For example, if the dopants are too deep or too shallow (on the surface), they will behave as recombination centers and thus reduce the overall solar to hydrogen efficiency [171]. The selection of deposition techniques for TiO_2_ structures will have an effect on the performance as these techniques have advantages and disadvantages. For example, chemical vapor deposition (CVD) and physical vapor deposition (PVD) produce homogenous and flexible microstructure and super hard materials, but the drawbacks are challenges with deposition rate, maintenance cost, and the size of the component. Thermal spraying has the advantage of fast deposition rates, large components, and ease of exploitation, but does not produce coatings of the same quality as CVD, electrodeposition, or PVD [172]. In other words, there is always the dilemma of choosing the correct deposition method and figuring out how it could affect the performance of the material. The research community employs a variety of techniques to characterize the material, infrared spectroscopy, Raman spectroscopy, scanning electron microscopy (SEM), X-ray spectroscopy, etc. Because the reviewed studies employ different characterization methods to verify, for example, the bandgap of TiO_2_ structures, a comparison of results in different studies becomes challenging. Another challenge is that experiments are not carried out under standardized conditions (e.g., with constant irradiance and homogeneous light distribution), the assessment of the real progress achieved with modified TiO_2_ is often difficult. Furthermore, comparisons between pristine TiO_2_ and enhanced photocatalysts are frequently biased because samples selected as reference materials present a relatively low photoactivity [173]. 

Computational modeling and simulations can help relieve some of these issues, although it comes with its own limitations. One advantage of theoretical simulations is that it is possible to create the exact structure you want to work on. Thus, we can investigate specific attributes and properties by fine-tuning the structure and composition of the compound. Moreover, the calculation of the H_2_ and O_2_ production is not based on measurement conditions, which makes results easier to compare. 

### 4.5. Production Facilities

**Figure 10:**
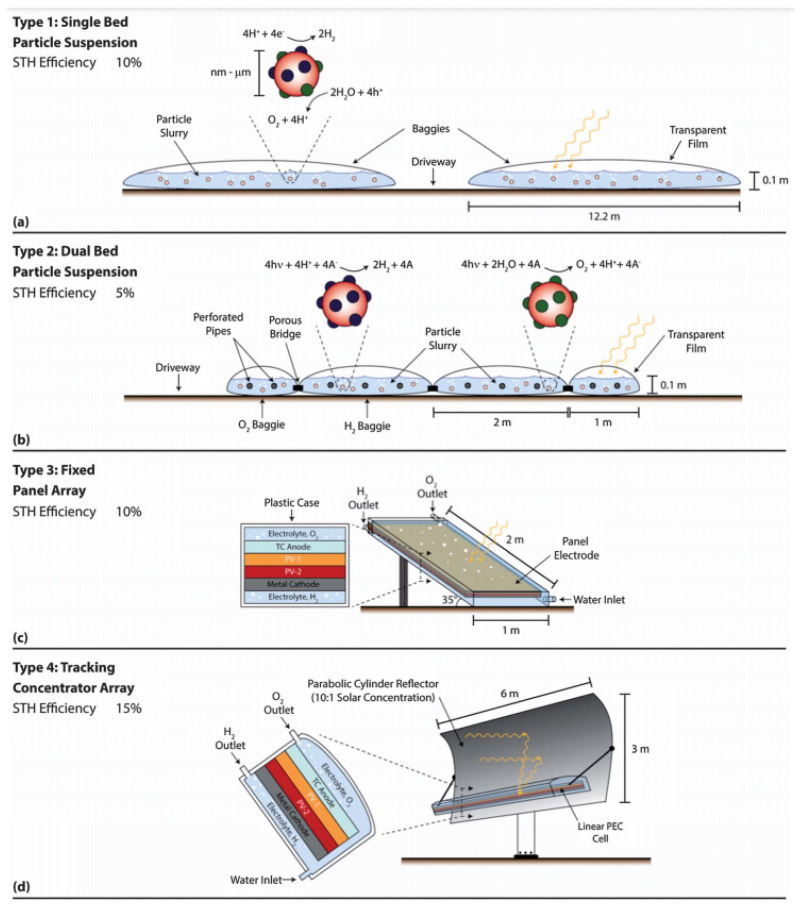
Schematic of the four different reactors. (**a**) Cross section of the Type 1 reactor showing the particle slurry contained by the baggies and separated by the driveway, (**b**) Type 2 reactor cross section with separate oxygen and hydrogen baggies connected by a porous bridge, (**c**) Type 3 reactor utilizing a photoelectrochemical (PEC) cell instead of photocatalytic water splitting (PWS) being directed toward the sun and (**d**) Type 4 reactor design that combines the PEC cell with an offset parabolic solar concentrator. Drawing not to scale. Reused with permission from [175]. Copyright 2013, with permission from Royal Society of Chemistry.

The aforementioned challenges are not the only ones that the research community faces in using TiO_2_ as the photocatalytic material for water splitting. Unfortunately, there is still a lack of scalable systems that could produce hydrogen in an economically feasible manner, even with an efficient catalyst [174]. Pinaud et. al. proposed and discussed the economic feasibility of four different designs of photocatalytic water splitting plants and the schematics are shown in Figure 10 [175]. 

Type 1 is a single bed particle suspension reactor and it is the simplest of the four. It consists of a low-lying horizontal plastic bag containing a slurry of photoactive particles in an electrolyte. The plastic bag is designed to allow light to penetrate, while it retains the electrolyte, photoactive particles, and evolved gases.

The Type 2 reactor is a dual bed with particle suspension, and it is similar to that of the Type 1 reactor. However, the biggest difference is that separate beds are used for H_2_ and O_2_ production.

The third option, named Type 3 reactor, is a fixed panel array, which consists of an integral planar electrode with multiple photoactive layers sandwiched between two electrodes. The entire system is within a transparent plastic electrolyte reservoir. The final alternative is the Type 4 reactor, which is a tracking concentrator array that uses an offset parabolic cylinder array to focus sunlight on a linear PEC cell receiver and has two-axis steering to track the daily movement of the sun [175].

In general, it was found that the key component for realizing these designs was to improve the solar to hydrogen efficiencies [175]. However, there are also other limitations, such as safety issues with the H_2_ and O_2_ gas mixture, how to split and collect the H_2_ and O_2_ gasses, a lack of general understanding of how the photocatalyst particle works, the mechanical integrity of the plastic bags, etc. [175]. 

## 5. Conclusions and Long-Term Outlook

Even though considerable progress has been made in the development of solar-driven water splitting with TiO_2_ as the photocatalyst, we believe there are four major challenges the research community must tackle before it becomes a viable technology.

The first challenge is the lack of a standard way to express the hydrogen production rates with varying photocatalytic materials. Research groups have been presenting these generations’ rates in different ways that make the comparison challenging. As seen in the literature presented here, the measured hydrogen evolution rates depend on specific details of the experimental setup, such as the spectrum of the light source, the light intensity at the sample, co-catalyst selection, size of the potential, and type and selection of the solution. Suggestions and solutions for standard experimental setups are also needed. However, reporting the apparent quantum yield (AQY) instead of only the gas evolution could be a part of the solution [176,177]. This will help bridge the gap between experimental and theoretical results. Computational modeling has some of the same challenges as the model, assumptions, approximations, and software used will affect the numerical results that make the comparison of results demanding. The missing piece here is calculations of the hydrogen and oxygen evolution rates, and an alternative can be studying the Volmer reaction, the Tafel reaction or the Herovsky reaction, and the Gibbs free energy [178,179].

The second challenge is the material TiO_2_ and its wide bandgap of 3.2 eV, which is in the ultraviolet section of the visible light spectrum. This means that 97% of the energy coming from the sun is not usable for TiO_2_ photocatalysts. There have been several attempts to lower the bandgap of TiO_2_, both experimentally and theoretically, which have been successful. However, the most successful experimental works are based on complex nanostructures or layered structures that are difficult and expensive to create at a larger scale. Computational modeling has in general focused more on various dopants and doping percentages. Unfortunately, the best results are seen when using noble metals or expensive metals. However, sulfur doping could be a solution to this problem. Combined TiO_2_ with other earth-abundant materials as MoS_2_ or WS_2_ could be better photocatalysts in the future [180,181].

The third challenge is the lack of research combining experimental research with theoretical simulations to optimize the characteristics of TiO_2_ structures for photocatalytic applications. This is a weakness in the current research as theoretical modeling and simulation could work as a great screening tool for the experimentalists, reducing their workload. Theoretical research could also help with the fundamental understanding of the process involved in solar-driven water splitting. By combining the two methods, it is easier to see the inner workings of the photocatalyst and to determine where improvement is needed. Moreover, computational work requires experimental verification and for validating the numerical results.

The final challenge is the lack of scalable systems that could produce hydrogen in an economically feasible manner, even with an efficient catalyst [174]. There is currently a lack of scalable and functional production facilities. The ones that do exist have a solar-to-hydrogen efficiency of 1.8% [182], indicating that further research is needed before photocatalytic water splitting is competitive with other hydrogen production methods. The main factor for the low efficiencies reported for photocatalytic hydrogen production is the low solar-to-hydrogen rates of the photocatalyst itself. An interesting idea is to look into combining TiO_2_ with perovskites due to the latter’s excellent optoelectronic properties. Naturally, water-insoluble perovskites combined with TiO_2_ could be the missing link that solar-driven water splitting needs.

## Figures and Tables

**Table 1:** This table displays the theoretical bandgap values for different doped TiO_2_ materials and for a few of these the H_2_ production rate is presented if published.

Nanomaterial	Bandgap [eV]	Ref.
Ag doped TiO_2_	2.312	[88]
Au doped TiO_2_	0.996	[88]
Pt doped TiO_2_	0.754	[88]
Pd doped TiO_2_	0.363	[88]
Ru doped TiO_2_	0.176	[88]
Wet TiO_2_ (001)	1.8571	[89]
Pt doped Wet TiO_2_ (001)	1.4546	[89]
Ru doped Wet TiO_2_ (001)	0.1636	[89]
Co doped Wet TiO_2_ (001)	0.0539	[89]
Ru clusters on TiO_2_	NA	[90]
Anatase TiO_2_	3.05	[91]
Pt adsorbed on TiO_2_	3.06	[91]
Pd adsorbed on TiO_2_	3.05	[91]
Rh adsorbed on TiO_2_	2.80	[91]
Ru adsorbed on TiO_2_	3.10	[91]
Anatase TiO_2_	2.98	[92]
Rutile TiO_2_	2.78	[92]
aTiO_2_	NA	[93]
Cu + N co-doped TiO_2_	NA	[94]
Cu doped anatase TiO_2_ (101)	NA	[95]
Cu doped anatase TiO_2_ (101)	NA	[15]
Co-doped SrTiO_3_	3.07	[96]
N-doped aTiO_2_	2.25	[97]
S-doped anatase TiO_2_	2.33	[98]
Nb-doped anatase TiO_2_	2.25	[98]
(S, Nb)-doped anatase TiO_2_	2.15	[98]
TiO_2_ hollow spheres doped with Mg	NA (H_2_ production rate: 850 µmol/h/g. O_2_ production rate: 425 µmol/h/g)	[99]
TiO_2_ doped by lanthanides	NA	[100]
C@O-doped TiO_2_	3.019	[102]
C@gap-doped TiO_2_	3.021	[102]
Nd@Ti-doped TiO_2_	3.032	[102]
Nd@gap-doped TiO_2_	2.353	[102]
C@O&Nd@Ti-doped TiO_2_	2.372	[102]
C&Nd@gap-doped TiO_2_	2.850	[102]
TiO_2-X_	2.6 (H_2_ production rate: 46.9 µmol/h/g)	[103]
g-CS@TiO_2-X_	2.5 (H_2_ production rate: 255.2 µmol/h/g)	[103]
g-CS+TiO_2-X_	2.3 (H_2_ production rate: 68.3 µmol/h/g)	[103]
Se(IV) ion doped TiO_2_	2.85	[104]
N-doped TiO_2_	3.06	[105]
Co-doped TiO_2_	2.92	[105]
Co-1N-doped TiO_2_	2.91	[105]
Co-2N-doped TiO_2_	2.90	[105]
Co-3N-doped TiO_2_	2.92	[105]
Mesoporous carbonate-doped phase-junction TiO_2_ nanotubes	2.69-2.92 (H_2_ production rate: 6108 µmol/h/g)	[106]
B-doped TiO_2_	2.40	[108]
S-doped TiO_2_	2.23	[108]
C-doped TiO_2_	2.53	[108]
P-doped TiO_2_	2.30	[108]
N-doped TiO_2_	2.51	[108]
F-doped TiO_2_	2.61	[108]
Cl-doped TiO_2_	2.34	[108]
N-TiO_2_	2.94	[109]
Cu-TiO_2_	3.22	[109]
(Cu, N)-TiO_2_	2.96	[109]
TiO_2_/g-C_3_N_4_	2.34	[109]
N-TiO_2_/g-C_3_N_4_	2.31	[109]
Cu-TiO_2_/g-C_3_N_4_	2.23	[109]
(Cu, N)-TiO_2_/g-C_3_N_4_	2.26	[109]
g-C_3_N_4_/TiO_2_	2.21	[110]
(C, N)-doped rutile TiO_2_	2.59	[111]
Rh, Nb co-doped TiO_2_	NA	[112]
S, N, or S+N doped TiO_2_ anatase (101) nanotubes	2.78–4.32	[113]
S-doped TiO_2_	2.72	[114]
Sc-doped three-layer fluorite structured TiO_2_	4.00	[115]
V-doped three-layer fluorite structured TiO_2_	3.95	[115]
Cr-doped three-layer fluorite structured TiO_2_	3.98	[115]
Mn-doped three-layer fluorite structured TiO_2_	3.66	[115]
Fe-doped three-layer fluorite structured TiO_2_	3.39	[115]
Co-doped three-layer fluorite structured TiO_2_	4.01	[115]
Ni-doped three-layer fluorite structured TiO_2_	4.20	[115]
Cu-doped three-layer fluorite structured TiO_2_	4.20	[115]
Zn-doped three-layer fluorite structured TiO_2_	3.60	[115]
4d metals doped TiO_2_ nanotubes	2–4	[116]
Three-layer TiO_2_ (101) nanotubes	3.83	[117]
Six-layer TiO_2_ (101) nanotubes	4.17	[117]
Nine-layer TiO_2_ (001) nanotubes	3.95	[117]
Six-layer TiO_2_ (001) nanotubes	4.15	[117]
Facer dependency of TiO_2_	NA	[118]
TiO_2_	NA	[119]
Phase stability in TiO_2_	NA	[120]
Rutile TiO_2_	NA	[121]
aTiO_2_	2.70–2.85	[14]

## Data Availability

No new data were created or analyzed in this study. Data sharing is not applicable to this article.
